# Dr. Brenan, on the Cure of Puerperal Fever

**Published:** 1816-12

**Authors:** John Brenan

**Affiliations:** 25, French Street, Stephen's Green, Dublin, October 16, 1816


### *To the Editors of the Medico-Chirurgical Journal & Review.*

GENTLEMEN,

IT is now about four years since I attempted the cure of Puerperal Fever in the Lying-in Hospital of Dublin. To introduce into medical practice a medicine possessing all the qualities that writers on materia medica ascribe to the spirits of turpentine, was a matter of innovation very arduous, but to cure by that innovation the Puerperal Fever, a disease hitherto mortal in nineteen cases out of twenty in its first stage, and invariably fatal in its every after stage, was to occasion a surprize in the medical world, that I could wish had produced in those professing the Heailng Art, more joy than dejection.

In my private practice as a physician in Dublin, I have saved the lives of thousands since that period by the judicious exhibition of the spirits of turpentine, as adjumentary to other medicines in various diseases; and I have had the satisfaction to find many worthy men amongst my medical brethren, who have adopted my practice with success and grateful acknowledgments. I shall take the liberty to mention a few facts, with respect to some puerperal cases that I have treated, in conjunction with other gentlemen, who can attest the facts I advance. Mrs. Connolly, the wife of a respectable gentleman in the law department, living in Queen Street, had been safely delivered on Monday by Dr. Clarke. On Thursday she was seized with violent rigors, and her abdomen became extremely sensitive. Dr. Clarke was sent for and informed, that she had puerperal fever; the Doctor happened to be indisposed, and Dr. Wall was called to see her. Dr. Wall was alarmed at the disease, never having seen any cases of it cured but those by me in the Lying-in Hospital; he ordered the abdomen, which was intolerably sensitive, to be rubbed with the spirits of turpentine, but never having seen it exhibited internally, declined further interference until I was called to see her. I found her in great pain all over the region of the uterus, unable to move without great distress. Her stomach much disordered, and her pulse above 100. She observed, that the rubbing of the turpentine, which had taken place about two hours before, had relieved her much; and, although she was then very uneasy, she observed that she felt much relieved by the external application of the medicine. I directed a draught, consisting of three drachms of spirits of turpentine, to be taken in some cinnamon water, with the same quantity of tincture of senna, and to be repeated every three hours; this was about twelve o'clock. Dr. Wall and I saw her in the evening; she had taken but one draught, and had three or four copious discharges from the bowels; her abdomen was, though sensitive, free from pain on pressure; her pulse was soft, and at 80 strokes in the minute; and she thought herself much relieved. I considered it unnecessary to visit her again; but under the care of Dr. Wall, and Mr. Halyday, an eminent apothecary, she progressively mended, and was quite well in a few days.

Case 2. I received directions to go to New Street, to see a young woman of delicate habit, who had been delivered of her first child, about ten days before, by Mr. Fitzsimons, an accoucheur of much experience, and who had continued to attend her up to that period; he informed me, that about four days previous to my coming, she sickened in the same manner as those affected with puerperal fever. I found her pulse low, and tremulous; her countenance was pallid; the abdomen swollen, and exquisitely sore on pressure. He informed me, that she had taken some purgative medicines, which answered their object, but with no relief to the pains she felt. I ordered the abdomen to be rubbed with spirits of turpentine, and I gave her a table spoonful of that medicine undiluted; in about fifteen minutes she expressed a sense of warmth all over her, and I found her pulse rose in fulness, though not in frequency. I desired that in an hour after, she should take an ounce of castor oil and an ounce of the spirits of turpentine, which produced before evening several black and copious evacuations; the abdomen could now, at eight o'clock, be pressed gently with only a sense of uneasiness; whereas, before she had been rubbed, it was intolerably pained on the slightest touch; and, to use the terms of the nurse, it was so distended as to resemble in appearance the glaze of a mahogany table. I directed that she should take an opiate, which gave her a quiet night; and in the morning, I repeated the castor oil and spirits of turpentine as before. These produced a few stools; and in the evening, she felt herself so well as to take some tea. I left her with directions to observe quietness; and on coming the next morning, I found her pains had returned, having, as I learned, taken a cold drink of porter. I had recourse to the same means as before, of applying the spirits of turpentine to the abdomen; and I gave half an ounce of it by injection in half a pint of the decoct. com. pro enem. The next morning she was much relieved, though much weakened. However, the symptoms of the disease which alarmed her, had nearly vanished; and by cautious treatment, in about eight days, she was able to come down stairs, and her recovery was progressive.

I am, &c.

JOHN BRENAN,* M. D.

25, French Street, Stephen's Green,
Dublin, October 16, 1816.

---

### *CASE of ENLARGED LIVER.*

* In our address to Correspondents last month, this name was by mistake spelt *Breume*.

